# Ultrasound-guided HIFU for uterine fibroids of hyperintense on T2-weighted MR imaging with or without GnRH-analogue-pretreated: A propensity score matched cohort study

**DOI:** 10.3389/fsurg.2022.975839

**Published:** 2022-08-04

**Authors:** Li Jiang, Jing-Wen Yu, Mei-Jie Yang, Qiao Zhong, Jin-Yun Chen

**Affiliations:** ^1^ State Key Laboratory of Ultrasound in Medicine and Engineering, College of Biomedical Engineering, Chongqing Medical University, Chongqing, China; ^2^ College of Medical Informatics, Chongqing Medical University, Chongqing, China; ^3^ Ultrasound Ablation Center, First Affiliated Hospital of Chongqing Medical University, Chongqing, China

**Keywords:** uterine fibroid, ablation, high-intensity focused ultrasound (HIFU), gonadotropinreleasing hormone analogue (GnRH-a), magnetic resonance imaging (MRI)

## Abstract

**Objective:**

To compare the therapeutic effect of high-intensity focused ultrasound (HIFU) ablation and HIFU pretreated with gonadotropin-releasing hormone analogue (GnRH-a) in the treatment of hyperintense uterine fibroids on T2-weighted magnetic resonance imaging (T2WI) by using propensity score matching.

**Materials and methods:**

339 women with 368 hyperintense uterine fibroids on T2WI who underwent single-session HIFU ablation were enrolled, including 283 patients with 303 fibroids in the single-session HIFU (sHIFU) group and 56 patients with 65 fibroids in the HIFU pretreated with GnRH-a (Gn-HIFU) group. The signal intensity (SI) value and standard deviation (SD) value were measured based on T2WI, and the fibroids were further subdivided into heterogeneous hyperintense fibroids, slightly homogeneous hyperintense fibroids and markedly homogeneous hyperintense fibroids as 3 subgroups (HHF, sHHF and mHHF group respectively). Treatment time, sonication time, dose, non-perfused volume (NPV), NPV per sonication time, non-perfused volume ratio (NPVR), energy effect ratio (EEF) and adverse events were recorded.

**Results:**

Out of 339 patients, the median NPVR was 75.2% (interquartile range,31.5%). After propensity score matching, the matched cohort included 91 (64.5%) patients in the sHIFU group and 48 (34.5%) patients in the Gn-HIFU group. The NPVR of sHHF in the Gn-HIFU group had significantly smaller than that in the sHIFU group (60.2% versus 74.9%, *p* = 0.005), and the NPVR of HHF in the Gn-HIFU group was higher than those in the sHIFU group (87.4% versus 72.9%, *p* = 0.002).

**Conclusions:**

Compared with HIFU alone, the therapeutic efficacy of the heterogeneous hyperintense fibroids may be enhanced by GnRH-a pretreated with HIFU, however it is important to rule out the slightly homogeneous hyperintense fibroids.

## Introduction

Uterine fibroids (UFs) are common benign neoplasms in female reproductive organs (1). UFs can be treated with medical therapy and surgical treatment, surgical treatment includes hysterectomy, myomectomy, uterine artery embolization (UAE) and high-intensity focused ultrasound (HIFU) ablation (2, 3).

HIFU ablation is to directly focus the ultrasound beam on the tumor, causing instant coagulative necrosis (1–3 s) in the target area (2, 4). The characteristic of the target medium is the basic condition of ultrasonic energy deposition. Magnetic resonance imaging (MRI) is the most accurate imaging technique for diagnosis and localization of UFs due to its excellent resolution of soft tissue and ability to reflect histological features (5). Funaki et al. reported the UFs were classified into three types based on the signal intensity of T2-weighted magnetic resonance imaging (T2WI), and the results indicate that hyperintense fibroids are more difficult to be treated than hypointense fibroids and isointense fibroids (6). Moreover, some studies showed that hyperintense fibroids could also be ablated with more ultrasonic energy than other signal intensity fibroids (7, 8). Recently, a study proposed that the signal intensity on T2WI was the most important factor affecting ablative efficiency (9). Therefore, whether HIFU ablation is suitable for hyperintense fibroids on T2WI and which treatment protocol should be adopted has become a research hotspot.

Gonadotropin-releasing hormone analogue (GnRH-a) is a synthetic derivative of gonadotropin-releasing hormone, with a function similar to that of GnRH *in vivo*, and has been applied for the management of UFs (10– 12). GnRH-a is supposed to improve the treatment efficacy of HIFU ablation by reducing the blood supply to fibroids and enhance the tissue responsiveness to thermal energy (13). To date, it has not been reported whether GnRH-a is effective in improving the response of hyperintense fibroids to HIFU ablation, and whether the response is different in hyperintense fibroids with different signal intensity. Therefore, the objective of this study is to compare the therapeutic effect of HIFU alone and HIFU pretreated with GnRH-a in the treatment of hyperintense fibroids on T2WI.

## Materials and methods

### Patients

This study was a retrospective analysis of consecutive women with UFs who underwent a single-session ultrasound-guided HIFU ablation treatment between January 2015 and January 2020 in our hospital. The study protocol was approved by the Ethics Committee at our institution (IRB: 2021-007) on August 31, 2021. All procedures followed were in accordance with ethical standards and the Declaration of Helsinki.

The inclusion criteria were as follows: (1) premenopausal patients over 18 years old; (2) patients with no more than three fibroids; (3) the maximum diameter of fibroids was at least 5 cm; (4) patients with pre- and post-HIFU MRI scanning, and MR scanning parameters were consistent.

The exclusion criteria were as follows: (1) patients with the presence of concomitant adenomyosis;(2) women who had a history of myomectomy; (3) patients who ever participated in other sex hormonal modulator therapy within 6 months before HIFU; (4) patients with significant degenerative fibroids assessed by enhanced MRI; (5) patients with a special category of fibroids, such as FIGO type 0, type 7 and type 8 (14); (6) patients with scar in the acoustic pathway, causing significant acoustic attenuation on the ultrasound scanning (sound attenuation width ≥10 mm).

### MRI evaluation and classification

All patients were assessed by pelvic contrast-enhanced MRI (CE-T1WI), MRI was performed with a 3.0-T MRI system (Singa HD Excite, GE Healthcare, USA). The MRI sequence is as follows: sagittal and axial T1W fast spin-echo (FSE): time of repetition (TR) 270 ms, time of echo (TE) 2.1 ms, layer thickness 5 mm, slice gap 1 mm; sagittal and axial T2W fast-recovery fast spin-echo (FRFSE): TR 3400 ms, TE 110 ms, layer thickness 5 mm, slice gap 1.5 mm; sagittal, coronal and axial CE-T1WI liver acceleration volume acquisition (LAVA): TR 4.2 ms, TE 2.0 ms, layer thickness 2.5 mm, slice gap 0.5 mm. An intravenous mass injection of 15–20 ml contrast agent gadodiamide (0.5 mmol/ml, Omniscan) was administered at 2 ml/s, and CE-T1WI was observed until 120s after injection.

UFs on T2WI were classified by quantitatively detecting the signal intensity (SI) value and standard deviation (SD) value of fibroids (13). The DICOM format of the preoperative MRI files was imported into the MicroSea-HIFU treatment 3D image assistance system (Chongqing MicroSea Software Development Co., Ltd., Chongqing, China). The largest three slices of fibroids on sagittal T2WI were selected to outline the region of interest (ROI) for automatically achieving SI value and SD value of each slice (15). The average SI value and SD value from the three slices were recorded, and the SD value indicated the signal homogeneity of fibroids. SI values of myometrium and endometrium were measured by the same method, and the SI values of uterine fibroids were compared for the fibroid classification.

**Figure 1:**
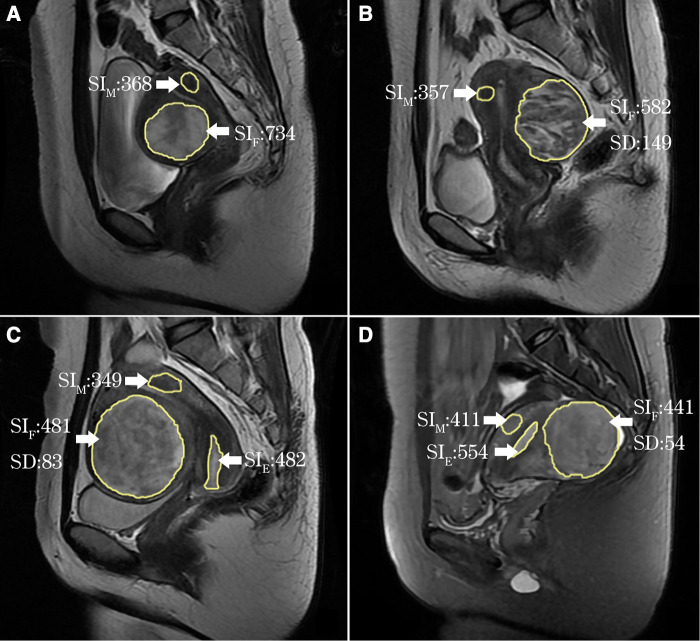
ROI delineation and measured value output on T2WI by the software (yellow line). (**A**) Uterine fibroids and myometrium ROI delineation and SI value output: SI_F_ > SI_M_; (**B**) HHF: SD = 149, SI_F_ ≥ SI_M_; (**C**) mHHF: SD = 83, SI_F_ ≈ SI_E_ > SI_M_; (**D**) sHHF: SD = 54, SI_M_ ≤ SI_F_ < SI_E_.

Hyperintense fibroids on T2WI were defined as the SI value of fibroids greater than or equal to the SI value of myometrium (SI_F_ ≥ SI_M_), and were classified into three types: (1) heterogeneous hyperintense fibroids (HHF): the signal of fibroids are heterogeneous, and SD ≥ 100 (**Supplementary Figure S1**, **Supplementary Table S1**); (2) markedly homogenous hyperintense fibroids (mHHF): fibroids SD < 100, the SI value of fibroids higher than that of the myometrium, and approximately equal to that of the endometrium (SI_E_ ≈ SI_F_ > SI_M_); (3) slightly homogenous hyperintense fibroids (sHHF): fibroids SD < 100, the SI value of fibroids higher than or equal to that of the myometrium, but lower than that of the endometrium (SI_E_ > SI_F_ ≥ SI_M_) (Figure 1).

### GnRH-a therapy protocol

GnRH-a has been a prescribing advice to manage large fibroids or with severe symptoms before HIFU ablation. GnRH-a (Leuprorelin, Beijing Biote Pharmaceutical Co., Ltd of China) was administered 3.75 mg subcutaneously starting on the 1st to 5th day of the menstrual cycle and thereafter every 4 weeks for 3 months. HIFU ablation was required to be completed within 28 days after administration of the last injection of GnRH-a.

### HIFU ablation

HIFU ablation was performed by HIFU-licensed physicians with at least 3 years of HIFU clinical experience using a Focused Ultrasound Tumor Therapeutic System (Model-JC, Chongqing Haifu Medical Technology Co., Ltd., Chongqing, China). The ultrasound transducer worked at a frequency of 0.5–1.5 MHz, and energy was adjustable in the range 0–400 W. Circulating degassed water was used as the coupling medium and the focal region was 1.5 × 1.5 × 10.0 mm. All patients received diet preparation, cleansing enema and skin preparation (shaving, degreasing and degassing) before treatment. A urinary catheter was inserted into the bladder and degassed normal saline was filled in to regulate the bladder volume during the procedure. The patients were positioned prone on the HIFU table, with the anterior abdominal wall in contact with degassed water. In order to establish an adequate safe acoustic pathway, an adjustable degassed water balloon was placed between the abdominal wall and transducer when necessary, to compress and push away the bowel from the acoustic pathway. HIFU ablation was performed under real-time ultrasonographic imaging guidance, the ultrasonic sonication time and acoustic power were adjusted according to the patient tolerance, the therapeutic energy was adjusted based on the changes in gray scale on guided ultrasonography. The sonication was terminated when the increased gray scale covered the planned ablation area, and sonication time was controlled within 3500s. Fentanyl-midazolam was intravenously used to keep patients conscious sedation, all patients were asked to report any discomfort during and after the procedure.

### Assessment of therapeutic response

**Figure 2:**
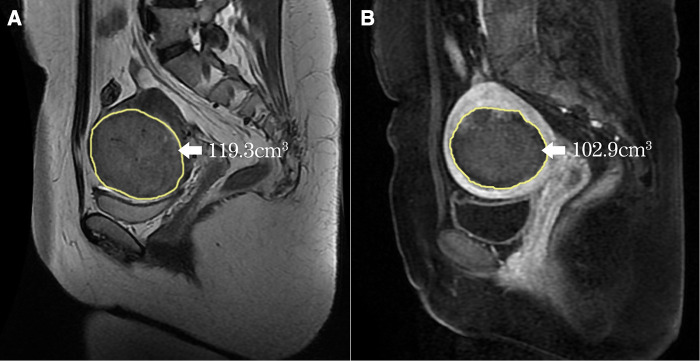
Measurement of the volume of the fibroid and the NPV (yellow line). (**A**) The fibroid was outlined on every slice of MRI, the automatically generated volume was 119.3 cm^3^; (**B**) The NPV was also outlined on every slice of CE-T1WI, the automatically generated NPV was 102.9 cm^3^.

Post-operative contrast-enhanced MRI within one week was used to evaluate the therapeutic effectiveness. Non-perfused volume (NPV) was measured on the CE-T1WI using software, the target volume was outlined slice-by-slice to generate the volume automatically (Figure 2). NPV ratio (NPVR) was calculated with the following equation: $NPVR=\frac{NPV}{Fibroid\;volume}\times 100\%$. NPV per sonication second (mm^3^/s, the fibroid necrosis volume in 1s sonication) was used to evaluate the efficiency of HIFU ablation. Energy effect ratio (EEF) was calculated with the following equation: $EEF=\frac{\eta \cdot Pt}{V}$, *η* was focusing factor (=0.7), P was average power (W), t was sonication time, V was NPV.

### Statistical analysis

The patients were divided into two groups: the sHIFU group and the Gn-HIFU group, according to whether GnRH-a-pretreated protocol was received. Propensity score matching was used to reduce the baseline differences between the two groups of the same sub-type, including the following parameters: age, body mass index (BMI), uterine volume and fibroids volume. The matched cohort was generated using 1:2 nearest neighbor propensity score matching, that one patient in the Gn-HIFU group was matched with two patients in the sHIFU group of the same sub-type. SPSS version 26.0 (IBM, Armonk, NY, USA) was used for statistical analysis. Normally distributed data were reported as the mean ± SD (standard deviation), non-normally distributed data were reported as medians and interquartile range. The student's t test, Mann–Whitney U test, one-way ANOVA, Kruskal–Wallis test, the chi-square and Fisher's exact tests to compare baseline, treatment time, sonication time, dose, NPV, NPV per sonication second, NPVR, EEF, and adverse events in patients with different sub-types of hyperintense fibroids on T2WI. When P value was less than 0.05, the difference was considered statistically significant.

## Results

### Patients

A total of 339 patients with 368 hyperintense fibroids on T2WI were enrolled. The mean age was 39.3 ± 7.1 (21–54) years, BMI was 22.6 ± 2.8 (15.4–32.9) kg/m^2^, the median uterine volume was 258.6 (207.8) cm^3^, the median fibroids volume was 76.3 (86.3) cm^3^. 303 (82.3%) patients who underwent single HIFU ablation were enrolled into the sHIFU group. 65 (17.7%) patients who underwent GnRH-a-pretreated before HIFU ablation were enrolled into the Gn-HIFU group, of these,14 patients underwent HIFU ablation after only 2 injections of GnRH-a.

### Ultrasound ablation results

All patients received HIFU ablation as planned, the median sonication time was 1,222 (819) s, the median treatment time 104.5 (60.0) min, the median dose was 476.8 (325.3) ×10^3^J, the median NPVR was 75.2% (31.5%), and the median EEF was 5.1 (7.3) J/mm^3^ (Table 1).

**Table 1:** Ultrasound ablation results.

Variable	Total
Number of fibroids	368
Power (W)	400 (9)
Treatment time (min)	104.5 (60.0)
Sonication time (s)	1222 (819)
Dose (×10^3^ J)	476.8 (325.3)
NPV (cm^3^)	51.3 (71.2)
NPV per sonication second (mm^3^/s)	52.7 (72.1)
NPVR (%)	75.2 (31.5)
EEF (J/mm^3^)	5.1 (7.3)

Note: Data are median value; interquartile range in brackets.

### Adverse events

Major adverse events occurred in one patient with a second-degree skin burn, which healed in 7 days after topical care. Minor adverse events included abdominal pain (51.9%), lumbar and back (sacrum) pain (44.2%), numbness and pain in lower limb (21.5%), buttock pain (14.57%), pain and distension of anus (1.5%), vaginal pain (0.3%). All patients recovered within 7 days (Table 2).

**Table 2:** Adverse events of ultrasound ablation.

Adverse event	Data (*n* = 339)
Minor adverse event	
Abdominal pain	176 (51.9)
Lumbar and back (sacrum) pain	150 (44.2)
Numbness and pain in lower limb	73 (21.5)
Buttock pain	50 (14.7)
Pain and distension of anus	5 (1.5)
Vaginal pain	1 (0.3)
Major adverse events	
Second-degree skin burn	1 (0.3)

Note: Data are given as *n* (%).

### Patients after cohort matching and ultrasound ablation results

After propensity score matching, a total of 139 patients with solitary fibroids were included in the matched cohort: 91 patients of the sHIFU group (38 patients with HHF, 18 patients with sHHF and 35 patients with mHHF); 48 patients of the Gn-HIFU group (20 patients with HHF, 10 patients with sHHF and 18 patients with mHHF). The baseline characteristics of matched cohort were well balanced. There was no significant difference in age, BMI, uterine volume and fibroid volume between the sHIFU group and the Gn-HIFU group (*p* > 0.05).

In the propensity score-matched cohort, there was no significant difference in power, treatment time, sonication time, dose, NPV, NPV per sonication second, EEF and NPVR between the sHIFU group and the Gn-HIFU group (*p* > 0.05) (Table 3).

**Table 3:** Comparison of baseline and ultrasound ablation results between the sHIFU group and the Gn-HIFU group after cohort matching.

Variable	sHIFU group (*n* = 91)	Gn-HIFU group (*n* = 48)	*p* value
Age (years)	38.2 ± 6.8 (22–51)	37.9 ± 6.7 (26–48)	0.666
BMI (kg/m^2^)	22.5 ± 3.0 (17.6–30.5)	22.0 ± 2.6 (16.6–27.3)	0.941
Uterine volume (cm^3^)	222.4 (166.9)	204.0 (199.9)	0.372
Fibroid volume (cm^3^)	67.4 (61.8)	76.6 (83.2)	0.339
Power (W)	400 (21)	400 (28)	0.226
Treatment time (min)	98.0 (62.5)	114.5 (63.5)	0.380
Sonication time (s)	1192.0 (893)	1236.0 (756.7)	0.505
Dose (×10^3^ J)	457.0 (367.3)	487.0 (299.6)	0.511
NPV (cm^3^)	46.8 (56.4)	61.4 (85.5)	0.487
NPV per sonication second (mm^3^/s)	45.9 (54.6)	50.7 (68.6)	0.557
NPVR (%)	74.3 (27.7)	75.0 (40.9)	0.886
EEF (J/mm^3^)	5.6 (6.7)	5.5 (7.6)	0.682

Note: Data are mean ± SD or median value; range or interquartile range in brackets.

### Comparison of results in different sub-types between the two groups

**Figure 3:**
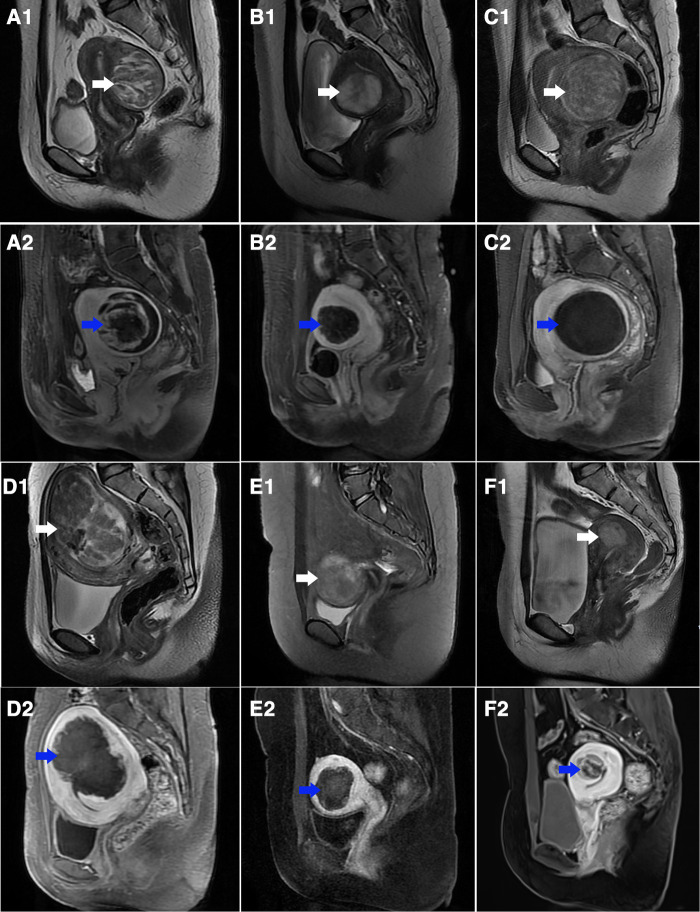
T2-weighted sagittal MR images of different sub-types of hyperintense fibroids before treatment and contrast-enhanced MR images after treatment. (**A1–C1**) T2-weighted images in the sHIFU group before treatment (white arrow): (**A1**) heterogeneous hyperintense; (**B1**) markedly homogeneous hyperintense; (**C1**) slightly homogeneous hyperintense; (**A2–C2**) contrast enhanced MR images after treatment counterpart to **A1**, **B1**, **C1** (blue arrow), the NPV is showed inside uterine fibroids: (**A2**) NPVR = 88.7%; (**B2**) NPVR = 68.9%; (**C2**) NPVR = 98.3%. (**D1–F1**) T2-weighted images in Gn-HIFU group before treatment (white arrow): (**D1**) heterogeneous hyperintense; (**E1**) markedly homogeneous hyperintense; (**F1**) slightly homogeneous hyperintense; (**D2–F2**) contrast enhanced MR images after treatment counterpart to **D1**, **E1**, **F1** (blue arrow), the NPV is showed inside uterine fibroids: (**D2**) NPVR = 76.2%; (**E2**) NPVR = 58.5%; (**F2**) NPVR = 27.7%.

The Gn-HIFU group and the sHIFU group's treatment outcomes for the same sub-type of hyperintense fibroids on T2WI were compared. The power, treatment time, sonication time, and dose for the heterogeneous hyperintense uterine fibroids were 400.0 W, 98.0 min, 1255.0 s, and 491.2 × 10^3^ J; 400.0 W, 128.0 min, 1296.5 s, and 527.2 × 10^3^ J, respectively. Power, treatment time, sonication time, and dose were not statistically different between the two groups; however, the Gn-HIFU group's NPV and NPVR were significantly greater than those of the sHIFU group (103.1cm^3^ vs 63.5 cm^3^, 87.4% vs 72.9%; *p* = 0.046, *p* = 0.002, respectively).Among the markedly hyperintense fibroids, the power, treatment time, sonication time and dose between the two groups were 400.0 W, 103.0 min, 1443.5 s and 573.3 × 10^3^ J; 393.5 W, 106.0 min, 1262.5 s and 496.6 × 10^3^ J, respectively. There was no difference between the two groups in terms of power, treatment time, sonication time, dose, NPV, NPV per sonication second, EEF, or NPVR (*p* > 0.05). Among the slightly hyperintense fibroids, the power, treatment time, sonication time and dose between the two groups were 400.0 W,95.0 min,1029.0 s and 358.0 × 10^3^ J; 399.0 W,103.5 min,1008.0 s and 391.4 × 10^3^ J, respectively. There was no difference in power, treatment time, sonication time, dose and NPV between the two groups, but the NPVR in the Gn-HIFU group was significantly lower than that in the sHIFU group (60.2% vs 76.7%, *p* = 0.005) (Figure 3, Table 4).

**Table 4:** Comparison of ultrasound ablation results in different sub-types of hyperintense uterine fibroids on T2WI between the sHIFU group and the Gn-HIFU group after cohort matching.

Groups	Number (*n*, %)	NPV (cm^3^)	NPVR (%)	NPV per sonication second (mm^3^/s)	EEF (J/mm^3^)
HHF group					
sHIFU	38 (27.3)	63.5 (80.0)	72.9 (34.7)	53.7 (59.7)	4.6 (6.5)
Gn-HIFU	20 (14.4)	103.1 (85.5)	87.4 (21.6)	64.8 (79.8)	4.3 (4.7)
*p* value		0.046^*^	0.002^*^	0.347	0.409
mHHF group					
sHIFU	18 (12.9)	48.4 (45.9)	71.2 (28.1)	37.9 (45.3)	7.5 (9.3)
Gn-HIFU	10 (7.2)	63.3 (63.3)	70.7 (49.0)	43.5 (85.5)	6.6 (10.1)
*p* value		0.759	0.796	0.555	0.588
sHHF group					
sHIFU	35 (25.2)	45.5 (43.0)	74.9 (22.1)	45.2 (39.0)	5.6 (5.7)
Gn-HIFU	18 (12.9)	25.0 (52.1)	60.2 (42.7)	45.3 (69.9)	6.2 (22.9)
*p* value		0.087	0.005^*^	0.639	0.560

Note: Data are median value; interquartile range in brackets.

* p < 0.05, Mann–Whitney *U* test.

## Discussion

HIFU ablation as an entirely non-invasive therapy, due to its excellent efficacy and safety for 195 symptomatic UFs, is a well-recognized treatment option (16). Prior to HIFU ablation, MRI has become a significant prediction and assessment tool based on imaging pathological features. The signal intensity on T2WI is the most important indicator influencing the efficiency of HIFU ablation for UFs (9).The signal intensity on T2WI, which can directly represent the properties of the tissue and to some extent reflect the kind of pathological lesions, can be used to diagnosis the histological subtypes of UFs. Funaki et al. classified UFs as three types including hypointense, isointense and hyperintense based on the signal intensity of T2WI, and reported that hyperintense fibroids should not be treated with HIFU by comparing the therapeutic effect and follow-up results of HIFU ablation for these three types of uterine fibroids (17– 19). According to research by Huang et al., hyperintense fibroids on T2WI have more cellular components, fewer collagen fibers, fluid-rich content, and abundant vascularization, all of which are unfavorable to the deposition of energy (20).

NPVR is the most important factor affecting the clinical outcome of HIFU ablation, and in HIFU ablation of UFs, achievement of an immediate NPVR of at least 80% is safe, with greater tumor volume shrinkage and better efficacy (21, 22). Due to technological improvement, NPVR ≥ 90% has been reported (23). In the present study, the median NPVR was 75.2% in 339 patients with hyperintense fibroids after HIFU ablation, and no serious adverse events occurred during treatment. It shows that, with current HIFU technology and clinical practice, it is challenging to obtain an NPVR of 80% for hyperintense fibroids on T2WI.

Pretreatment with GnRH-a was first reported in 2006 to enhance the thermal effect of HIFU ablation (24), Funaki et al. also suggested the combination therapy with GnRH-a may improve the treatment effect of HIFU ablation for hyperintense fibroids (17). GnRH-a, as one of the drug treatments for uterine fibroids, was observed to cause shrinkage in fibroids size and reduction of blood supply in fibroids (13, 25).It has been proven the crucial part that estrogen/progesterone receptor (ER/PR) expression plays in the development of UFs, which are benign tumors dependent on ovarian hormones (26, 27). The expression of ER and PR in UFs has been demonstrated to decrease in response to GnRH-a (28). The likelihood of fibroids recurrence would also be increased if the remnant fibroid cells expressed ER and PR strongly because HIFU ablation is an in-tumor treatment and would undoubtedly result in cellular residues. Due to more postoperative residual lesions compared to other signal intensity fibroids following HIFU ablation, the rates of recurrence or reintervention were higher in hyperintense fibroids on T2WI (19, 29). It could be inferred that HIFU pretreated with GnRH-a may reduce the recurrence rate of UFs. In this study, the therapeutic effects of HIFU ablation and HIFU in combination with GnRH-a in the treatment of hyperintense fibroids on T2WI were compared using propensity score matching, and the results showed that there was no significant difference between the two groups. However, the NPVR of hyperintense fibroids after GnRH-a pretreatment with HIFU was 75%, which was significantly higher than that of symptomatic fibroids in previous studies (13).

In hyperintense fibroids on T2WI, there are still different histologic manifestations. Based on the classification of hyperintense fibroids by Zhao et al (8), our study proposed a quantitative classification method using MR values that could further categorize hyperintense fibroids into 3 sub-types by quantitatively detecting the SI and SD values on T2WI, it could be more precise and repeatable than experience-based visual typing. The efficacy of each sub-type between HIFU treatment and HIFU combined with GnRH-a treatment was compared, and the results showed that the NPVR of HH fibroids was ≥80% after the combined treatment, which was significantly higher than that of HIFU treatment. However, the combination treatment resulted in a much lower NPVR for sHHF (60.2%) than the HIFU treatment. Our findings indicated that, while GnRH-a pretreatment should be avoided for sHHF, it can be utilized to improve the efficacy of HIFU ablation for HHF in hyperintense fibroids on T2WI, which are challenging to ablate.

It is important to note that some patients required HIFU ablation after only two injections of GnRH-a therapy and did not finish the three-injection regimen as intended. The majority of patients in randomized controlled research at week four after leuprorelin medication did not exhibit clear symptoms of estrogen deficiency, but after nine weeks of leuprorelin treatment, the incidence of adverse events was much higher (30). Therefore, it is speculated that some of the study's participants had low tolerance and were unable to handle the discomfort of estrogen insufficiency after receiving two doses of GnRH-a treatment, as a result of which they refused to receive the third GnRH-a injection. Compared with the efficacy of single-dose GnRH-a pretreatment combined with HIFU ablation for UFs (13), two-dose and three-dose GnRH-a were more effective. And two-dose GnRH-a may be the appropriate dose to improve efficacy with better compliance, however, this requires further investigation in clinical studies.

Skin burns are a common complication of HIFU treatment, especially in patients with surgical scars on the lower abdomen. In this study, patients with sound attenuation width ≥10 mm caused by abdominal scar were excluded, which effectively reduced the occurrence of skin burns. Scar patch could be used safely and efficiently in MRgHIFU treatment for patients with lower abdominal surgical scars (31), whether it can be used in patients with sound attenuation of more than 10 mm deserves further study. In this study, neither the sHIFU group nor the Gn-HIFU group experienced any major or life-threatening adverse events related to the treatment. Only one case of superficial second-degree skin burn occurred in the Gn-HIFU group, the patient was an overweight woman (BMI 25.6 kg/m^2^), due to the excessive sonication time and dose delivery, which were 3,030 s for the sonication time and 1,147,250 J for the total dose, the temperature increased in the nearfield of the ultrasound beam path by excessive energy deposition during the HIFU ablation and would ultimately result in skin burns (32). After the active dressing change, the patient's skin lesions healed without scar formation. As a result, the safety of using GnRH-a in combination with HIFU to treat hyperintense fibroids on T2WI has also been established. However, as greater doses were still needed, dose prediction and safety assessment were necessary prior to ablation.

Our study is limited because it is a retrospective study and it is unable to intervene in the course of GnRH-a treatment, there were fewer cases in the Gn-HIFU group than in the sHIFU group, which made comparisons between the two groups subject to bias. Propensity score matching, however, can more effectively reduce the bias. To support these results, additional prospective trials are required. Long-term clinical follow-up is required to determine if GnRH-a in combination with HIFU ablation could impact the clinical presentation and the reintervention rate of different subtypes of hyperintense fibroids on T2WI. Medical costs are also a common concern of both doctors and patients. GnRH-a is used as a pretreatment before HIFU in the Gn-HIFU group, in terms of treatment approach, the Gn-HIFU group has incurred higher medical expenses than the sHIFU group. However, clinical effectiveness is a determinant of cost-effectiveness (33). For the heterogeneous hyperintense fibroids, the Gn-HIFU group has higher efficacy than the sHIFU group, indicating that these patients will benefit more from the treatment. The results of this study also have practical health economics value for avoiding the use of GnRH-a in patients with ineffective subtypes. Certainly, prospective studies with follow-up of long-term outcomes and more detailed cost-benefit analyses are warranted.

## Conclusions

In conclusion, based on our retrospective propensity score matching cohort study results, the therapeutic efficacy of the heterogeneous hyperintense fibroids may be enhanced by GnRH-a pretreated with HIFU, however it is important to rule out the slightly homogeneous hyperintense fibroids. It might serve as a reference for clinical HIFU ablation for uterine fibroids treatment and offer patients and physicians a better therapeutic option.

## Data Availability

The data analyzed in this study is subject to the following licenses/restrictions: The datasets presented in this article are not readily available because the data contains the patient's private information, it cannot be used publicly. Requests to access these datasets should be directed to Jin-Yun Chen, chenjy@cqmu.edu.cn.
