# Supplementary material for: Ultrasound-guided HIFU for uterine fibroids of hyperintense on T2-weighted MR imaging with or without GnRH-analogue-pretreated: A propensity score matched cohort study
Source: Front Surg. 2022 Aug 4;9:975839. doi: 10.3389/fsurg.2022.975839 (PMC9386137; doi:10.3389/fsurg.2022.975839)
Supplement: Supplementary file 1 [file Table_1_v1.docx]

Supplementary Material

# Supplementary Figures and Tables

## Supplementary Figures


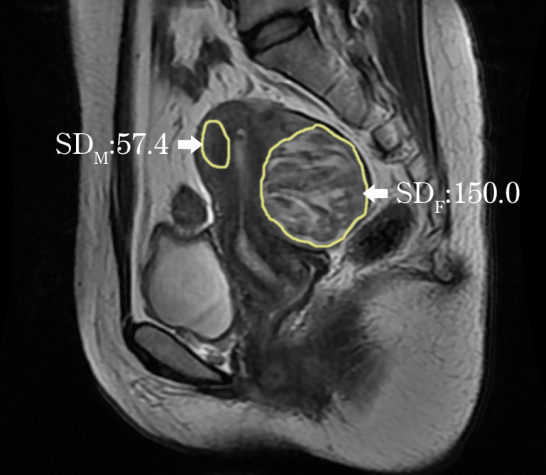


**Supplementary Figure 1.** ROI delineation and measured value output on T2WI by the software (yellow line). SD_M_, SD value of the myometrium, SD_M_=57.4; SD_F_, SD value of the heterogeneous hyperintense fibroids, SD_F_=150.0.

- 1. **Supplementary Tables**

The largest three slices of fibroids on sagittal T2WI were selected to outline the region of interest (ROI) for automatically achieving SD value of each slice. The average SD value from the three slices were recorded.

**Supplementary Table 1.** Reference ranges of SD values in heterogeneous hyperintense fibroids.

| Parameters | Number | Median | Reference ranges |
| --- | --- | --- | --- |
| SD_M_ | 40 | 55.4(46.4,67.6) | 31.4-93.3 |
| SD_F_ | 40 | 142.5(116.0,167.6) | 101.1-223.0 |

Note: SD_M_, SD value of the myometrium; SD_F_, SD value of the heterogeneous hyperintense fibroids. Reference ranges are presented as 2.5th and 97.5th percentiles; interquartile range in brackets.
